# Hip fracture risk assessment: artificial neural network outperforms conditional logistic regression in an age- and sex-matched case control study

**DOI:** 10.1186/1471-2474-14-207

**Published:** 2013-07-15

**Authors:** Wo-Jan Tseng, Li-Wei Hung, Jiann-Shing Shieh, Maysam F Abbod, Jinn Lin

**Affiliations:** 1 Department of Orthopaedic Surgery, National Taiwan University Hospital Hsin-Chu Branch, No.25, Ln. 442, Sec. 1, Jingguo Rd., East Dist., 300, Hsinchu, Taiwan; 2 Department of Orthopaedic Surgery, National Taiwan University Hospital, No.7, Zhongshan S. Rd., Zhongzheng Dist., Taipei, Taiwan; 3 Department of Mechanical Engineering, Yuan Ze University, No.135, Yuandong Rd., Zhongli, Taiwan; 4 School of Engineering and Design, Brunel University, Kingston LaneUxbridge Middlesex UB8 3PH, West London, United Kingdom

**Keywords:** Hip fracture, Artificial neural network, Conditional logistic regression, Discrimination, Calibration

## Abstract

**Background:**

Osteoporotic hip fractures with a significant morbidity and excess mortality among the elderly have imposed huge health and economic burdens on societies worldwide. In this age- and sex-matched case control study, we examined the risk factors of hip fractures and assessed the fracture risk by conditional logistic regression (CLR) and ensemble artificial neural network (ANN). The performances of these two classifiers were compared.

**Methods:**

The study population consisted of 217 pairs (149 women and 68 men) of fractures and controls with an age older than 60 years. All the participants were interviewed with the same standardized questionnaire including questions on 66 risk factors in 12 categories. Univariate CLR analysis was initially conducted to examine the unadjusted odds ratio of all potential risk factors. The significant risk factors were then tested by multivariate analyses. For fracture risk assessment, the participants were randomly divided into modeling and testing datasets for 10-fold cross validation analyses. The predicting models built by CLR and ANN in modeling datasets were applied to testing datasets for generalization study. The performances, including discrimination and calibration, were compared with non-parametric Wilcoxon tests.

**Results:**

In univariate CLR analyses, 16 variables achieved significant level, and six of them remained significant in multivariate analyses, including low T score, low BMI, low MMSE score, milk intake, walking difficulty, and significant fall at home. For discrimination, ANN outperformed CLR in both 16- and 6-variable analyses in modeling and testing datasets (p?<?0.005). For calibration, ANN outperformed CLR only in 16-variable analyses in modeling and testing datasets (p?=?0.013 and 0.047, respectively).

**Conclusions:**

The risk factors of hip fracture are more personal than environmental. With adequate model construction, ANN may outperform CLR in both discrimination and calibration. ANN seems to have not been developed to its full potential and efforts should be made to improve its performance.

## Background

With increased human life expectancy, osteoporosis has become more prevalent and may lead to disastrous fractures at most skeletal sites. Among them, hip fractures are of particular concern because they are associated with a significant morbidity (functional recovery being limited to less than 50% [1]) and excess mortality (up to 18-33% in the first year and persisting for at least 5 years afterwards [2]). The number of hip fractures worldwide is projected to hit 2.6 million by 2025 and may rise to 4.5 million by 2050, imposing huge health and economic burdens upon societies as a whole [3]. For developing strategies to prevent this serious injury, it is of crucial importance to better understand its risk factors and identify the patients at risk. Although many potential risk factors contributing to hip fracture have been identified, such as low bone mineral density (BMD), old age, female gender, chronic health conditions, experience of fracture and falls, physical inactivity, heavy smoking and drinking, impaired vision, use of certain medicines, low calcium and vitamin intake, low body mass index (BMI), low muscle strength, etc. [4,5], these risk factors may vary geographically, ethnically, and culturally, and their combined effects have not been well understood [6]. We have several kinds of method to create the risk factor models for hip fracture evaluation, and conditional logistic regression (CLR) and artificial neural network (ANN) are popular among them.

The ANN, simulating high-level human brain functions, is a computational modeling tool that has become widely accepted for modeling complex real-world problems [7]. Although it has been explored in many areas of medicine, such as nephrology, microbiology, radiology, neurology, cardiology, etc. [8], its use in the orthopedic trauma field is still rare. Eller-Vainicher et al. identified the promising role of ANN in predicting osteoporotic fracture among postmenopause osteoporosis women [9]. Lin et al. found ANN algorism could reliably predict the mortality of hip fractured patients and outperforms the logistic regression method [10]. The ANN, consisting of a set of highly interconnected processing units (neurons) tied together with weighted connections, includes an input layer, one or more hidden layers, and an output layer. The input layer comprises the data available for the analysis, and the output layer comprises the outcome. The ANN is trained on the basis of training data to correlate the input with the corresponding output over repeated training epochs to reduce the overall error. The stimulus of the input is propagated forward through each neuron layer until the output is produced. Then the ANN output is compared to the observed output, and an error signal is calculated. This error signal is then transmitted backwards across the neuron layers and the connection weights are updated to reduce the overall error. This refers to the multiplayer perceptron ANN model with feedforward backpropagation training and this process is supervised by a group of validation data, which are not used in the training process, and is terminated when the validation error reaches its minimum. ANN models derived from this training process are applied to other new datasets not used for training and validation.

In the present age- and sex-matched case control study, we identified important risk factors for hip fractures and the results were further used to build hip fracture prediction models with CLR or ANN methods. Based on a fair comparison with the same dependent variables and analytical processes, we hypothesized that ANN with a more nonlinear approach outperforms CLR in both discrimination and calibration.

## Methods

### Participants

The inclusion criteria were non-institutionalized patients over 60 years of age who had first-time, low-energy hip fractures, defined as fractures of the proximal femur caused by injuries equal to or less than a fall at standing height. Patients with previous hip lesions or surgeries were excluded. The study was approved by institute review board of National Taiwan University Hospital. Between April 2004 and January 2006, a total of 366 patients older than 60 years were admitted to our institute under the diagnosis of hip fractures. Among them, 115 cases were excluded for the following reasons: previous hip fractures or surgeries (76), fractures not caused by low-energy trauma (25), fractures in institutionalized patients (13), and the fracture treated without surgery (1). Of the 251 patients who met the inclusion criteria, 217 patients (149 women and 68 men) gave written informed consent and were enrolled in the current study. All patients were interviewed under stable conditions after their surgeries. The median time for completing the interview was 6 days after the fracture.

Hospital controls were simultaneously selected from patients of the Department of Family Medicine at the same hospital with the diagnosis of diseases or injuries unrelated to bone and without any history of hip fractures. The control group was individually matched to cases by age (within 4 to 6 years) and sex. Informed consents were obtained from all the participants.

### Data measurements

Selection of the risk variables was based on the results of previous studies and other potential causes of hip fracture in an older population. Both cases and controls were interviewed by trained interviewers with the same standardized questionnaire including questions on 66 variables in 12 categories: 1) socio-demography (six variables: ethnicity, education, occupation, marriage, income, and living arrangement); 2) disease history (14 variables: hypertension, diabetes, stroke, heart disease, chronic respiratory disease, arthritis, osteoporosis, liver disease, cancer, cataract, Parkinson’s disease, constipation, weakness, and headache or migraine); 3) self-assessed health (three variables: current, comparison with 1 year ago, and comparison with same-aged people); 4) anthropometry (three variables: height, weight, BMI); 5) health habits (three variables: smoking, alcohol consumption, and regular exercise); 6) diet habits and medicine (15 variables: vegetarian diet, intake of milk, coffee, tea, calcium, vitamin, glucosamine, or anti-hypertensive, other cardiovascular, analgesic, anti-diabetic, psychotropic, gastrointestinal, and other drugs, and multiple medications); 7) injury-related experience (four variables: history of fall-induced fractures, fracture location, significant fall at home in the past year, and history of fall outdoors); 8) environmental hazards (seven variables: building type, multistory dwelling, number of stairs in a flight, stair height, stair lighting, outdoor lighting, and green light duration near their home); 9) physical functions (four variables: Activities of daily living (ADL) difficulty; Instrumental ADL (IADL) difficulty, walking difficulty, and pain at walking); 10) cognitive and other functioning (five variables: urinary incontinence, fecal incontinence, vision, hearing, and Mini-Mental State Examination (MMSE) score); 11) coordination function; and 12) total BMD. Height and weight were measured using electronic scales for BMI calculation. The physical functions were measured by questions on the level of difficulty in performing five ADL (eating, bathing, dressing, transferring, toileting), six IADL (using the telephone, managing medications, preparing meals, maintaining the home, shopping, managing finances), and walking. Cognitive function was measured with the MMSE. The coordination function was measured by finger-to-nose test which was conducted by asking the participants to use their finger to alternately touch their own nose and the interviewers’ finger as quickly as possible. BMD (T-score) was examined at the non-fractured side of proximal femur for cases and the same side for matched controls by using the same machine of dual-energy x-ray absorptiometry (DEXA) (Model: QDR4500A; Hologic, Waltham, MA), and read by the same radiologist. The reliability of interview and measurement results among the interviewers was checked by intraclass correlation coefficient (ICC), which showed moderate to high agreement.

### Data processing and risk factor selection

The data were analyzed with conditional logistic regression to produce odds ratios and 95% confidence intervals using statistic software of SPSS COXREG 17.0 (SPSS Inc., Chicago, IL). Univariate analysis was initially conducted to examine the unadjusted association of all potential risk factors with hip fracture. Continuous variables including monthly income, body weight, height, leisure-time physical activity, MMSE score, peak expiratory flow rate, average hand grip strength, and total BMD value were all categorized into two groups according to the cut-off point selected by the Youden index in the receiving operating characteristic (ROC) curves. A “Missing” category was created for BMD with missing data. Significant variables with p?<?0.1 in univariate analyses were then tested by multivariate analyses with the forward stepwise approach, with the p value set at 0.05 for entry and 0.1 for removal. Categorical variables were contrasted with reference to the other category. All statistical tests performed were 2-tailed, and the final significance level was set at 0.05.

The significant variables in univariate and multivariate analyses were used to compute the individual fracture risk with either CLR or ANN. The dependent variable, hip fracture, was a dichotomous variable (Yes?=?1; No?=?0). All predictors were binary variables, coded with 0 or 1 (missing?=?2).

### Participant partition

**Figure 1:**
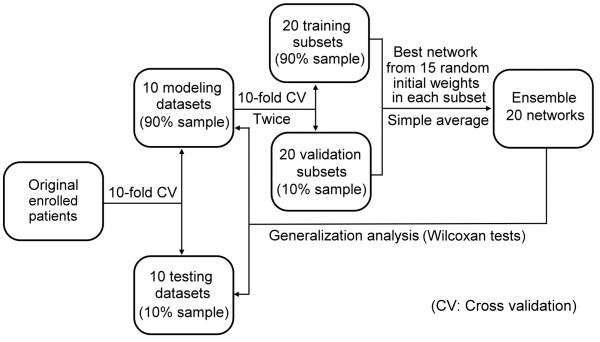
The flowchart of data partition, neural network creation and generalization analyses by cross validation.

To assess the generalization, three way data split method [11] (Figure 1) was used for construction of prediction models and internal cross validation. The 217 matched pairs were randomly divided into two separate groups for 10-fold cross validation analyses: 195–197 pairs (about 9/10 of the enrolled patients) as the modeling datasets and 20–22 pairs (about 1/10) as the testing datasets. The modeling group was used to build CLR and ANN models. The testing group was set aside for later tests for generalization.

### Conditional logistic regression model

In CLR analyses, the regression equations were derived from the significant variables in univariate and multivariate analyses in the modeling datasets. Risk scores calculated by regression equations as the summation of the products of the included independent variables and the regression coefficients of the variables were used to assess hip fracture risk [12]. The regression equations were then applied to the subjects in testing datasets for generalization analyses.

### Artificial neural network model

In ANN analyses, the participants in each modeling dataset were further randomly divided into two subsets: 9/10 as the training subsets and 1/10 as the validation subsets also based on the principle of 10-fold cross validation. This procedure was performed twice, and thus 20 groups of training and validation subsets were obtained for ensemble analyses. In the training subsets, feed-forward back-propagation neural networks consisted an input layer, hidden layers, and an output layer, were constructed. A scaled conjugate-gradient algorithm [13] was used as a supervised learning algorithm to train the network. It adjusted the internal weights and biases of the network according to the second-order gradient information over repeated training epochs to reduce the overall error. One epoch consisted of a single presentation of each set of inputs followed by automatic adjustments of the weight connections to minimize the total error for all data that were used in the training. The estimation of error was based on the mean-squared error. The parameter, which determined the change in the weight for the second derivative approximation (σ), was set to 5×10^-5^. The parameter, which regulated the indefiniteness of the Hessian (λ), was set to 5×10^-7^. A logistic transformation of the weighted inputs to the output node was applied to determine the overall output of the network, which would range from 0 to 1. The training was terminated if the error in the validation subsets stopped dropping or, indeed, started to rise (early stopping). The number of hidden neurons was determined according to the test running on 5 to 25. In each group of training and validation subsets, 15 sets of different initial weights were analyzed, and the networks with the lowest validation errors were selected. Thus we got 20 networks after the twice 10-fold cross validation training and validating each time and these 20 networks were combined to generate the ensemble models by simple average of the outputs. These ensemble models were applied to the testing datasets. The variables used in ANN analyses were the same as those in CLR analyses. The ANN analyses were run by Neural Network Toolbox in MATLAB 7.8 (R2009a, MathWorks, Natick, MA).

### Comparison of performance of models

In both modeling and testing datasets, the validity was checked by discrimination, and the reliability was checked by calibration (goodness of fit [14]). The discriminatory power of the models was assessed using the area under the ROC curves (AUROC). Discrimination refers to the ability to distinguish positive from negative cases. A good discriminating model in the present study would assign a higher risk score to hip fracture cases. Sensitivity, specificity, and accuracy were calculated in modeling and testing datasets according to the cut-off points selected by the Youden index on ROC curves. The calibration power of the models was compared using Hosmer-Lemeshow (HL) statistics [15]. The HL statistic is a single summary measure of the calibration and is based on comparing the observed and estimated fractured cases. The smaller the HL statistic is, the better the fit, with a perfectly calibrated model having a value of zero. Meanwhile, calibration curves based on the deciles from the data calculated using observed and expected values were built. The relationship between the observed and expected values was evaluated by ICC. The performance of classifiers, including discrimination, calibration and other measures of accuracy, sensitivity and specificity on the 10 pairs of ANN and CLR datasets, was compared using Wilcoxon signed-rank tests (p?<?0.05).

## Results

Of the 149 pairs of women and 68 pairs of men, the average age was 80.7?±?7.8 (mean?±?standard deviation) years for women and 80?±?7.4 years for men in the fracture group and 77.8?±?6.8 years for women and 78.4?±?7.9 years for men in the control group. In univariate analyses among the 66 variables, 16 variables achieved significant level (Table 1). Milk intake meant milk consumption at least six times a week. Walking difficulty meant inability to walk or walking with assistance of crutches or walkers. Significant fall at home meant major fall at home more than once in the past year. Low education level meant lower than junior middle school. Current smoking meant a smoking habit of more than half of a pack per day. Fecal incontinence meant experience of uncontrolled stool passage. Vision impairment was recorded according to patients’ subjective feeling of impaired vision during walking. ADL difficulty meant impairment of at least two of the five activities. IADL difficulty meant impairment of at least two of the six activities. Regular exercise meant exercise habit at least four times per week. Coordination abnormality meant under or over shooting of a target and impaired timing or integration of muscle activity during finger-to-nose examination. In multivariate analyses, six variables remained statistically significant (Table 1). BMD was the most important factor causing hip fractures with the highest odds ratio and statistical significance. The average T-score was much lower in hip fracture patients than that in controls, -2.58?±?1.06 vs. -1.85?±?1.3. It was also lower in women than in men (−2.8?±?1.02 vs. -1.9?±?0.92 for fractured patients and −1.6?±?1.18 vs. -0.6?±?0.93 for controls). Here we chose BMD alone to access its prediction ability for hip fractures with CLR analyses in order to compare its combined effects with other risk factors.

**Table 1:** Results of univariate and multivariate analyses of CLR

	**Control**	**Case**	**Crude OR**	**p-value**	**Adjusted OR**	**p-value**
	**n?=?217**	**n?=?217**	**(95% CI)**		**(95% CI)**	
BMD, T-score ≤-1.70	67 (30.9%)	117 (53.9%)	9.04 (4.46-18.3)	<0.001	8.11 (3.49-18.8) (3.49-18.83)	<0.001
missing	18 (8.29%)	73 (33.6%)	19.5 (8.19-46.6)		16.5 (5.62-48.5)	
BMI ≤21.4	76 (35.0%)	134 (61.8%)	2.78 (1.82-4.25)	<0.001	2.38 (1.18-4.76)	0.016
MMSE score ≤19	47 (21.7%)	115 (53.0%)	4.10 (2.42-6.61)	<0.001	2.66 (1.23-4.88)	0.008
Milk intake	136 (62.7%)	176 (81.1%)	0.36 (0.22-0.61)	<0.001	0.23 (0.09-0.57)	0.016
Walking difficulty	145 (66.8%)	179 (82.5%)	2.40 (1.40-4.10)	0.001	2.68 (1.12-6.20)	0.026
Significant fall at home in past year	23 (10.6%)	56 (25.8%)	3.39 (1.82-6.28)	<0.001	2.15 (1.24-5.4)	0.012
Low education level	31 (14.3%)	68 (31.3%)	2.43 (1.46-4.04)	0.001		
Current smoking	38 (17.5%)	45 (22.0%)	2.20 (1.04-4.65)	0.039		
Previous fractures after age 55 years	20 (9.20%)	45 (20.7%)	2.22 (1.27-3.88)	0.005		
Fecal incontinence	28 (12.9%)	48 (22.1%)	1.90 (1.09-3.30)	0.024		
Vision impairment	31 (14.3%)	50 (23.0%)	1.76 (1.05-2.94)	0.031		
<2 Major diseases	139 (64.1%)	110 (50.7%)	0.59 (0.39-0.87)	0.009		
ADL difficulty	26 (12.0%)	60 (27.6%)	2.59 (1.50-4.46)	0.001		
IADL difficulty	136 (62.7%)	161 (74.2%)	1.64 (1.01-2.67)	0.045		
Regular exercise	129 (59.5%)	99 (45.6%)	0.54 (0.35-0.83)	0.005		
Coordination abnormality	14 (6.45%)	42 (19.4%)	3.40 (1.68-6.88)	0.001		

**Figure 2:**
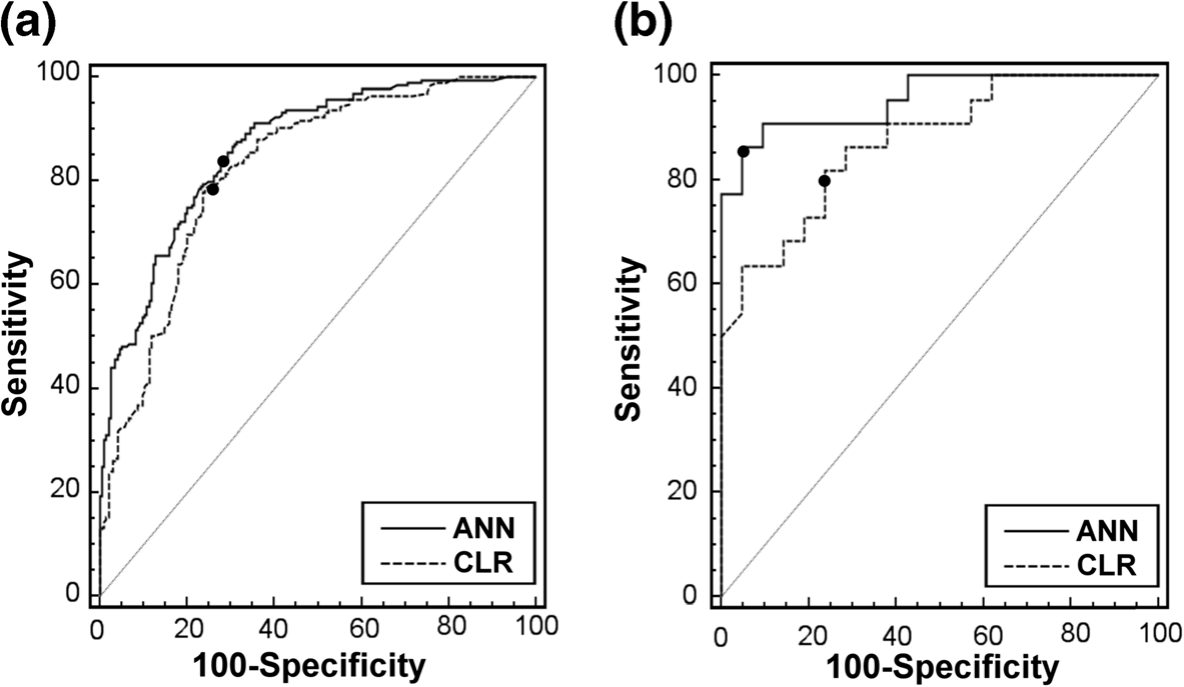
**Comparison of discrimination power. (a)** ROC curves in the modeling dataset. **(b)** ROC curves in the testing dataset. Black dots indicate the cut-off points determined by Youden Index.

**Figure 3:**
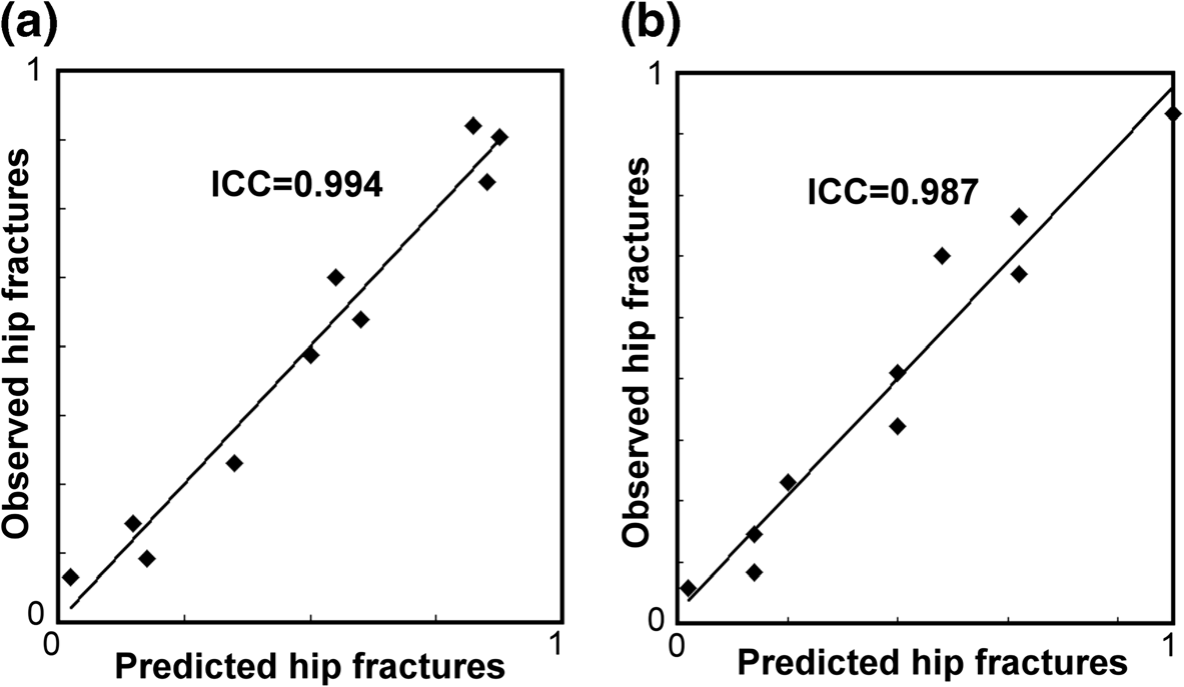
**Comparison of calibration power in modeling datasets. (a)** Calibration curves in ANN models. **(b)** Calibration curves in CLR models. Calibration curves were based on predictions determined by deciles.

**Figure 4:**
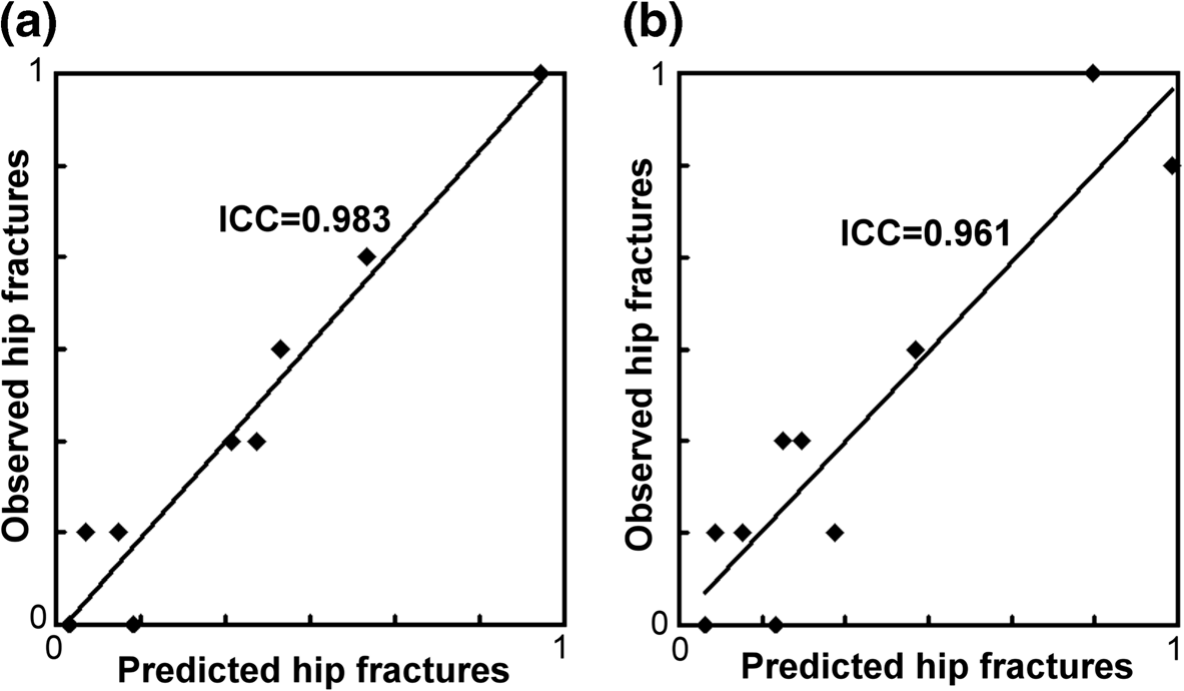
**Comparison of calibration power in testing datasets. (a)** Calibration curves in ANN models. **(b)** Calibration curves in CLR models. Calibration curves were based on predictions determined by deciles.

The neural network with eight hidden neurons was selected in the training process. For discrimination in modeling datasets, ANN was significantly higher than CLR in AUROC and accuracy in 16- and 6-variable models (Table 2) (Figure 2). The sensitivity was not significantly different in the two models. For specificity, ANN was significantly higher than CLR only in the 16-variable model. In testing datasets ANN was significantly higher than CLR in AUROC and accuracy in the 16- and 6-variable models. There was no significant difference for sensitivity and specificity. In some datasets, AUROC and accuracy were very close between ANN and CLR, e.g., testing datasets 3 (0.865 vs. 0.863) and 4 (0.807 vs. 0.801) in 6-variable models. The accuracy of CLR was even higher than that of ANN in testing dataset 6 (0.698 vs. 0.651) and 7 (0.698 vs. 0.697) in 6-variable models. As for calibration in modeling datasets, ANN had significantly lower HL Chi-squares and was more calibrated than CLR in 16-variable models (Table 3) (Figure 3). There was no significant difference in 6-variable models. ICCs were not significantly different in the two models. In testing datasets, ANN was more calibrated than CLR with significantly lower HL chi-squares and higher ICCs in 16-variable models. In 6-variable models, HL chi-squares were not significantly different, but ANN still had significantly higher ICCs (Figure 4).

**Table 2:** Discrimination of ANN and CLR in modeling and testing datasets with 16- and 6-variable models

	**1**	**2**	**3**	**4**	**5**	**6**	**7**	**8**	**9**	**10**	**Mean**	**SD**	**p**
Modeling													
AUROC													
ANN 16v	0.888	0.867	0.866	0.869	0.864	0.880	0.873	0.861	0.886	0.866	0.872	0.009	0.005*
CLR 16v	0.835	0.828	0.829	0.832	0.824	0.850	0.837	0.828	0.840	0.836	0.834	0.007	
ANN 6v	0.849	0.839	0.839	0.842	0.831	0.853	0.848	0.832	0.837	0.837	0.841	0.007	0.005*
CLR 6v	0.826	0.825	0.818	0.826	0.816	0.836	0.828	0.815	0.821	0.823	0.823	0.006	
Accuracy													
ANN 16v	0.805	0.790	0.790	0.785	0.785	0.810	0.805	0.785	0.815	0.805	0.797	0.011	0.005*
CLR 16v	0.769	0.763	0.761	0.771	0.769	0.781	0.774	0.774	0.778	0.768	0.771	0.006	
ANN 6v	0.770	0.761	0.760	0.786	0.775	0.780	0.775	0.760	0.770	0.765	0.770	0.008	0.005*
CLR 6v	0.753	0.746	0.746	0.769	0.763	0.766	0.758	0.751	0.758	0.753	0.756	0.008	
Sensitivity													
ANN 16v	0.790	0.760	0.800	0.800	0.860	0.820	0.830	0.780	0.820	0.790	0.805	0.027	0.444
CLR 16v	0.770	0.857	0.779	0.872	0.779	0.892	0.856	0.830	0.785	0.781	0.820	0.044	
ANN 6v	0.780	0.840	0.820	0.860	0.840	0.800	0.850	0.840	0.860	0.810	0.830	0.025	0.959
CLR 6v	0.724	0.704	0.867	0.888	0.872	0.887	0.882	0.876	0.728	0.796	0.822	0.072	
Specificity													
ANN 16v	0.820	0.820	0.780	0.770	0.710	0.800	0.780	0.790	0.810	0.820	0.790	0.032	0.012*
CLR 16v	0.767	0.668	0.742	0.668	0.758	0.670	0.691	0.718	0.772	0.755	0.721	0.041	
ANN 6v	0.760	0.680	0.700	0.710	0.710	0.760	0.700	0.680	0.680	0.720	0.710	0.028	0.368
CLR 6v	0.782	0.788	0.624	0.648	0.655	0.644	0.634	0.626	0.788	0.708	0.690	0.067	
Testing													
AUROC													
ANN 16v	0.815	0.894	0.905	0.890	0.955	0.792	0.876	0.948	0.773	0.836	0.868	0.059	0.005*
CLR 16v	0.769	0.773	0.853	0.825	0.872	0.721	0.824	0.891	0.707	0.772	0.801	0.059	
ANN 6v	0.806	0.878	0.865	0.807	0.908	0.777	0.842	0.948	0.838	0.866	0.854	0.048	0.005*
CLR 6v	0.778	0.793	0.863	0.801	0.845	0.758	0.810	0.904	0.817	0.800	0.817	0.041	
Accuracy													
ANN 16v	0.765	0.811	0.836	0.811	0.768	0.701	0.741	0.840	0.680	0.729	0.768	0.053	0.017*
CLR 16v	0.767	0.744	0.814	0.698	0.791	0.674	0.698	0.816	0.614	0.705	0.732	0.062	
ANN 6v	0.743	0.860	0.857	0.676	0.743	0.651	0.697	0.906	0.730	0.795	0.766	0.081	0.028*
CLR 6v	0.721	0.698	0.791	0.674	0.698	0.698	0.698	0.811	0.682	0.727	0.720	0.043	
Sensitivity													
ANN 16v	0.760	0.760	0.860	0.760	0.910	0.730	0.770	0.830	0.680	0.760	0.782	0.063	0.759
CLR 16v	0.857	0.857	0.864	0.762	0.818	0.727	0.773	0.783	0.591	0.762	0.779	0.077	
ANN 6v	0.810	0.860	0.950	0.620	0.860	0.680	0.770	0.960	0.820	0.900	0.823	0.104	0.575
CLR 6v	0.762	0.524	1.000	0.810	0.864	0.773	0.773	0.870	0.591	0.810	0.777	0.129	
Specificity													
ANN 16v	0.770	0.860	0.810	0.860	0.620	0.670	0.710	0.850	0.680	0.700	0.753	0.084	0.066
CLR 16v	0.682	0.636	0.762	0.636	0.762	0.619	0.619	0.850	0.636	0.652	0.685	0.075	
ANN 6v	0.680	0.860	0.760	0.730	0.620	0.620	0.620	0.850	0.640	0.700	0.708	0.087	0.202
CLR 6v	0.682	0.864	0.571	0.545	0.524	0.619	0.619	0.750	0.773	0.652	0.660	0.103	

* Statistically significant difference.

**Table 3:** Calibration of ANN and CLR in modeling and testing datasets with 16- and 6-variable models

	**1**	**2**	**3**	**4**	**5**	**6**	**7**	**8**	**9**	**10**	**Mean**	**SD**	**p**
Modeling													
Chi-square													
ANN 16v	5.791	10.735	6.784	12.737	5.859	4.315	6.067	9.698	5.161	6.981	7.413	2.583	0.013*
CLR 16v	18.458	19.948	9.761	9.008	9.222	12.553	10.714	18.406	10.518	17.758	13.635	4.221	
ANN 6v	9.077	6.323	6.482	9.398	7.679	3.729	6.560	10.786	8.217	6.973	7.522	1.877	0.333
CLR 6v	14.913	5.333	5.125	6.997	12.961	4.859	11.817	9.667	12.676	3.386	8.773	3.914	
ICC													
ANN 16v	0.994	0.989	0.991	0.984	0.992	0.994	0.992	0.99	0.993	0.995	0.991	0.003	0.066
CLR 16v	0.984	0.977	0.992	0.993	0.992	0.987	0.991	0.981	0.991	0.979	0.987	0.006	
ANN 6v	0.992	0.995	0.992	0.995	0.994	0.996	0.996	0.993	0.993	0.994	0.994	0.001	0.066
CLR 6v	0.986	0.992	0.996	0.995	0.985	0.994	0.989	0.98	0.988	0.998	0.990	0.005	
Testing													
Chi-square													
ANN 16v	9.365	4.227	7.363	6.317	6.281	5.044	9.150	2.706	7.778	6.576	6.481	1.984	0.047*
CLR 16v	8.618	6.884	15.622	8.691	9.798	6.046	4.914	7.248	8.566	12.228	8.862	2.968	
ANN 6v	7.334	7.647	8.936	2.493	8.714	15.144	14.947	2.043	8.193	3.678	7.913	4.309	0.646
CLR 6v	8.828	8.713	12.350	10.182	10.132	7.305	9.845	4.358	5.295	6.889	8.390	2.315	
ICC													
ANN 16v	0.927	0.985	0.976	0.979	0.975	0.944	0.952	0.995	0.942	0.966	0.964	0.021	0.007*
CLR 16v	0.906	0.944	0.912	0.949	0.952	0.883	0.965	0.965	0.907	0.922	0.931	0.027	
ANN 6v	0.957	0.966	0.965	0.976	0.961	0.883	0.874	0.996	0.942	0.983	0.950	0.039	0.037*
CLR 6v	0.926	0.915	0.936	0.929	0.933	0.888	0.893	0.979	0.955	0.961	0.932	0.027	

* Statistically significant difference.

For using BMD alone to assess the fracture risk by CLR in modeling datasets, the AUROC and HL chi-square were 0.723?±?0.01 and 17.21?±?4.523, respectively. In testing datasets, the AUROC and HL chi-square were 0.702?±?0.056 and 12.86?±?5.214. The discrimination and calibration of the model of BMD alone was lower than the model created by BMD and other risk factors in CLR model (Table 2, Table 3).

## Discussion

In the present study, univariate CLR analysis identified 16 significant factors, including low T-score, walking difficulty, low BMI, low MMSE score, low milk intake, significant fall at home, low education, smoking habit, fractures experienced after age 55 years, fecal incontinence, vision impairment, presence of major diseases, ADL difficulty, IADL difficulty, no regular exercise, and coordination abnormality. The first six factors remained statistically significant in stepwise multivariate analysis, with low T-score being the most important one among them. In comparison of ANN and CLR for fracture risk assessment, ANN provided statistically higher discrimination and calibration power in the modeling and testing datasets in cross validation analyses.

In the literature, various clinical risk factors have been reported for hip fractures [4], but their combined effects for fracture prediction varies. The present matched case control study investigated most of the different kinds of potential personal and environmental risk factors. The 16 significant factors left in univariate analysis were mostly personal and modifiable. This outcome supports the finding that at-home falls of old people are mainly due to impaired general health, rather than external hazards [16], and emphasizes the importance of improving bone strength and general health for fracture prevention. It has been reported that milk supplement can increase the bone density in Chinese women [17,18] and low milk intake could lead to high fracture risk in our study. Low milk intake might also account for low education level which was associated with high fracture risk [19]. Walking difficulty and low MMSE could account for vision impairment, poor coordination, low ADL and IADL. Low BMD, the most significant variables in our analyses, could account for smoking habit, associated diseases, lacking of exercise, fecal incontinence and previous fractures. BMD measurement is an important tool for assessing osteoporosis. It can be used for diagnosis, monitoring of treatment, and fracture risk prediction. Hip fracture risk increased by 3.7 times per SD decrease in femoral neck BMD at the age of 50 years [20]. The present study supports the finding that combining BMD and clinical risk factors can further improve the predictability of hip fracture and emphasize the multidirectional approach for patient at risks.

Logistic regression and ANN are currently the most widely used models for diagnosis and prognosis studies in biomedicine. Logistic regression has the advantages of high interpretability of model parameters and ease of use, but the use of linear combinations of variables is not suitable for modeling highly nonlinear complex interactions as is demonstrated in biologic and epidemiologic systems [21]. ANN with its resemblance to the human brain is appealing because of flexible nonlinear systems that show robust performance in dealing with noisy, incomplete or missing data and have the ability to generalize. They may be better at predicting outcomes when the relationships between the variables are multidimensional as found in complex biological systems. The ANN model allows inclusion of a large number of variables and there are not many assumptions (such as normality) that need to be verified. However, the comparative performance of these two methods has been widely reported with great controversy in the literature. In a review of 28 major studies carried out by Sargent [22], the performance was superior for ANN in 10 studies (36%), was superior for logistic regression in 4 cases (14%), and was similar in the remaining 14 cases. In another review of 72 papers conducted by Dreiseitl and Ohno-Machado [15], with statistical tests, both models performed similarly in 42%, ANN better in 18%, and logistic regression better in 1%. By contrast, without statistical tests, ANN was better in 33% and logistic regression better in 6%. The authors also surveyed the quality of the methodology and found a shortage of reporting ANN model building details in 49%, lack of statistical testing in 39%, and lack of calibration information in 75%. ANN is theoretically more flexible than logistic regression because of multi-layer networks, but on the other hand, it is threatened by over-fitting and instability [23]. Especially, there are still no set methods for constructing ANN models [23], which may lead to the wide variation in the comparative results.

Over-fitting ANN model which are trained too closely on limited available data would lose its generalization. The network with generalization could offer reasonable outputs in new unseen data. A commonly used method to improve generalization in data-mining is a three-way data split with cross validation [11] as in the present study. The modeling datasets were split into training and validation subsets. The error on the validation subset was monitored during training epoch and once the error had increased, the training was stopped (early stopping). The network with lowest validation errors was chose. This generalization property may obtain good output data without training on all possible available datasets. Another practical problem is ANN instability [23] which means that changes in the training data may produce very different models and consequently different performance on unseen data. The instability is caused by training getting caught in different local minima in the error surface. This instability problem can be fixed by building ANN ensembles and aggregating the results of the networks [24]. The aggregated outputs with diversified individual networks will have lower variance and smaller bias than a single network. Furthermore, the 10-fold cross splitting method used for building the ANN ensembles could ensure each datum was equally used for both training and validation. The present study showed that ANN significantly outperformed CLR in terms of discrimination and calibration in both 16- and 6-variable models. However, it may lead to biased superior performance in ANN training or validation subsets when compared with CLR models. Thus, we used the cross validation testing datasets for ANN and CLR generalization comparison. Besides, as shown in the Table 2, comparison of discrimination on a single testing dataset might lead to no significant difference or even higher accuracy in CLR. This might explain the high inconsistency in the comparisons of these two classifiers reported in the literature, especially if statistical testing was not performed [15,25]. In the present study, nonparametric tests for paired samples in 10 cross validation groups could detect the significant difference between the two classifiers in datasets with varied patterns.

Sensitivity, specificity and accuracy determined according to a pre-specified cutoff point are also commonly used for comparing the performance of the classifiers [15]. Actually, the risk score computed by the classifiers may be affected by the disease prevalence; thus selection of the cutoff points is important for a fair comparison. In the present study, the Youden index defined by the point with the minimum of the summation of the false positive and false negative rates in the ROC curve best differentiates between subjects with disease and those without disease when equal weight is given to sensitivity and specificity. Using the Youden index as the cut-off point can be independent from the disease prevalence and makes the predicting models more applicable to different series of patients [26]. It has been reported that the use of a cut-off point arbitrarily determined at a risk score equal to 0.5 might lead to biased results and unfair comparisons [27].

The present study had limitations. First, as a matched case control study, age and sex were not included in the predictive models. This exclusion might lower the performance of the classifiers. Second, some clinical risk factors were not included, such as the geometry of the proximal femurs or maternal history of hip fractures, because the former is not a routine examination for the elderly and the latter might be subject to information or reporting bias. Third, all the continuous variables were converted to binary variables with a cut-off point of the Youden index. This method could maximize the difference between cases and controls and make the comparison more fair and clinical application easier. However, some important information might be lost if the distribution of the variables was complex [28]. Fourth, it was not fair for CLR if the interaction terms or quadratic functions were not included. However, these interaction terms were not routinely examined in conventional analyses. Besides, no significant interaction between the input variables was found in the present study. Fifth, participant partition using 10-fold cross validation method in the present study might result in a sample size too small for validation and testing and increase the variance [25]. Besides, this sample size was also not enough for a standard HL analysis, which required at least 400 cases [29]. Bootstrap resampling method might be another option to improve the efficiency of validation. Last, although considerable efforts, through many trial-and-errors, were made to optimize the design of the neural networks, they still could be further improved in model topology or ensemble method [22].

## Conclusions

The hip fracture risk in the elderly can be effectively assessed by neural networks and logistic regression analyses. The risk factors identified in the present study are more personal than environmental. Combining BMD and clinical risk factors can predict the fracture risk better than BMD alone. With adequate model construction and comparison, ANN may outperform CLR in both discrimination and calibration. However, ANN seems have not been developed to its full potential. More studies to further improve its performance are warranted. The models created in this study still need to be validated externally.

## Abbreviations

CLR: Conditional logistic regression; ANN: Artificial neural network; BMD: Bone mineral density; BMI: Body mass index; ADL: Activities of daily living; IADL: Instrumental activities of daily living; MMSE: Mini-mental state examination; DEXA: Dual-energy x-ray absorptiometry; ICC: Intraclass correlation coefficient; ROC: Receiver operating characteristic; AUROC: Area under the ROC curve; HL: Statistics: Hosmer-Lemeshow statistics.

## Competing interests

The authors declare that they have no competing interests.

## Authors’ contributions

WJT contributed to study design, data analysis and drafted the manuscript. LWH contributed to data analysis and drafted the manuscript. JSS, MFA and JL contributed to study design and manuscript review. All authors read and approved the final manuscript.

## Pre-publication history

The pre-publication history for this paper can be accessed here:

http://www.biomedcentral.com/1471-2474/14/207/prepub
